# Validity and Reliability of Published Comprehensive Theory of Mind Tests for Normal Preschool Children: A Systematic Review

**Published:** 2015-09

**Authors:** Seyyede Zohreh Ziatabar Ahmadi, Shohreh Jalaie, Hassan Ashayeri

**Affiliations:** 1 Department of Speech & Language Pathology, School of Rehabilitation Sciences, Iran University of Medical Sciences, Tehran, Iran; 2 Biostatistics Division, School of Rehabilitation Sciences, Tehran University of Medical Sciences, Tehran, Iran; 3 Department of Rehabilitation Basic Sciences, School of Rehabilitation Sciences, Iran University of Medical Sciences, Tehran, Iran

**Keywords:** *Theory of Mind Test*, *Preschool*, *Normal*, *Validity*, *Reliability*

## Abstract

**Objective:** Theory of mind (ToM) or mindreading is an aspect of social cognition that evaluates mental states and beliefs of oneself and others. Validity and reliability are very important criteria when evaluating standard tests; and without them, these tests are not usable. The aim of this study was to systematically review the validity and reliability of published English comprehensive ToM tests developed for normal preschool children.

**Method:** We searched MEDLINE (PubMed interface), Web of Science, Science direct, PsycINFO, and also evidence base Medicine (The Cochrane Library) databases from 1990 to June 2015. Search strategy was Latin transcription of ‘Theory of Mind’ AND test AND children. Also, we manually studied the reference lists of all final searched articles and carried out a search of their references. Inclusion criteria were as follows: Valid and reliable diagnostic ToM tests published from 1990 to June 2015 for normal preschool children; and exclusion criteria were as follows: the studies that only used ToM tests and single tasks (false belief tasks) for ToM assessment and/or had no description about structure, validity or reliability of their tests. Methodological quality of the selected articles was assessed using the Critical Appraisal Skills Programme (CASP).

**Result:** In primary searching, we found 1237 articles in total databases. After removing duplicates and applying all inclusion and exclusion criteria, we selected 11 tests for this systematic review.

**Conclusion: **There were a few valid, reliable and comprehensive ToM tests for normal preschool children. However, we had limitations concerning the included articles. The defined ToM tests were different in populations, tasks, mode of presentations, scoring, mode of responses, times and other variables. Also, they had various validities and reliabilities. Therefore, it is recommended that the researchers and clinicians select the ToM tests according to their psychometric characteristics, validity and reliability.

Theory of mind (ToM) or mind reading is an aspect of social cognition that evaluates mental states and beliefs of oneself and ot hers (1-7). For the first time, Premack and Woodruff (1978) referred to theory of mind as the child's ability to explain thoughts, feelings and ideas of his/her own and others and to predict their behavior (1). For precise understanding of social cognition, we need to have a mature ToM. 

Development of ToM is hieratical. It begins with identification and expression of facial expression and follows by identification of false beliefs of oneself and others (8). For example, very young children (aged 1-2) can understand the first levels of ToM skills (identification of facial expression) and 3-5 year-old children can carry out more complex ToM skills (9-13). False belief tasks refer to the understanding that other people can have beliefs about the worlds that are different from their own. In other words, awareness of false beliefs allows children to understand, explain and predict the wrong actions of others (10, 12 and 14). Development of most difficult levels of ToM such as irony and humor understanding occurs in children over 6 years of age (10, 12 and 14).

The ToM assessment instruments are important for the identification of ToM difficulties and the evaluation of treatment progress in children with hearing loss (HL), specific language impairment (SLI), pervasive developmental disorder (PDD), and mental retardation (MR) (15). From 1980 up to now, many tests have been designed to assess ToM skills. They are different in models of presentation, tasks, time of execution, validity and reliability. We now are aware that the one important criterion for judging a test is its validity and reliability (15). In the past (1980-1990), most researchers have used just one single task measurement that included single aspects of ToM. These assessments may have been quick, but provided no information about other aspects of ToM and stability of ToM ability over time (15). We recognize that ToM includes not only false belief tasks but also other aspects (e.g., facial expression recognition, pretend plays) (15). Therefore, psychologists recommend the use of comprehensive instruments which contain multiple tasks (15). Such instruments can reduce standard errors and make measurements more reliable and valid. The total score of such a test is a compound score (15). Compound scores are stable, because they include multiple factors and lead to a more accurate measurement of the basic skills (16, 17). Therefore, we need to define these comprehensive ToM tests, particularly for normal preschool children. 

 ToM tests are important for predicting language and cognitive impairments. Although many studies have been conducted to assess ToM abilities in children, to date no study has been done to review and assemble validity and reliability of these ToM tests. This study collected all comprehensive published English ToM tests through a systematic review. This information may be used to help researchers and clinicians to choose more suitable published English ToM tests to evaluate social cognition.

## Material and Methods

***Searching the Literature***

We searched MEDLINE (PubMed interface), Web of Science, Science direct, PsycINFO, and also Evidence Base Medicine (The Cochrane Library) databases from 1990 to June 2015. The study population was normal preschool children. Search methods included the combination of text word field searching, using controlled vocabulary and a Boolean operator. Search strategy included Latin transcription of ‘Theory of Mind’ AND test AND children (Appendix 1). It was adapted to each database with minor changes. All the searches were conducted to obtain studies published in June 2015. Also, we manually studied the reference lists of the final articles as well and carried out a search of those references.

***Inclusion and Exclusion Criteria***

**Fig 1:**
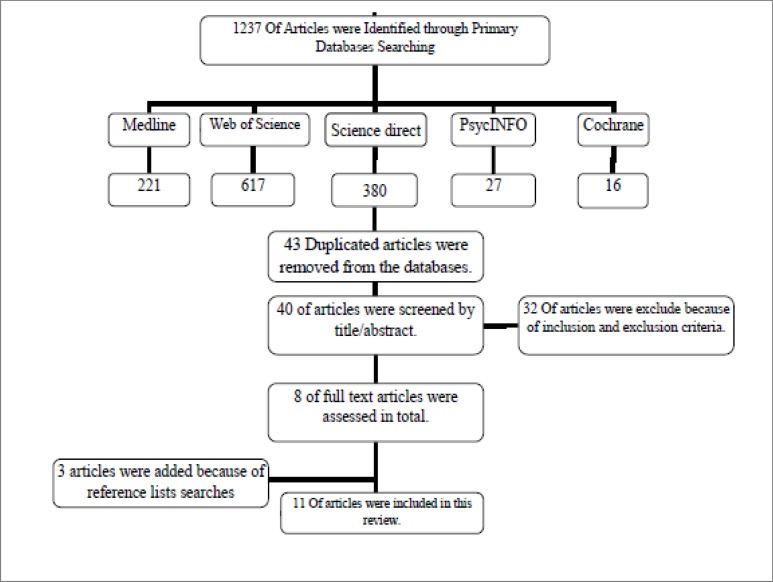
Process of Reviewing the Diagram

Articles were screened using inclusion and exclusion criteria detailed in Figure 1. Criteria for inclusions were those English articles published from 1990 to June 2015 that were relevant to ToM tests, and those studies done on normal preschool children; Criteria for exclusion were the articles that had insufficient information about ToM assessment or ToM tests in children, the articles that only used ToM tasks and did not have test development, and those articles that used single tasks (e.g., false belief tasks) to assess total ToM skills and/or had no description about structure and development of their tests. 

***Selecting and Screening the Studies***

Screening the studies was done by two researchers (SZZA, ShJ) in one day independently and verified by a third author from the research team. A total of 1237 articles were searched by the primary searching of all databases (Medline: 221; Web of Science: 617; Science direct: 380; PsycINFO: 27 and Cochrane: 16). Then, we removed articles that were not related to ToM test development or did not provide sufficient information about assessment of ToM in children; we selected 83 articles by searching the titles. After excluding 43 duplicated articles, 40 articles remained. We selected 8 of those articles after studying the title/abstracts and applying all inclusion and exclusion criteria. Then, we studied the full texts of the articles and manually searched the reference lists of 8 final articles and added 3 references to the searched articles. Thus, 11 articles were included in this systematic review (Fig 1). 

***Quality Assessment of Screened Studies***

Quality assessment was performed by each author from the research team. Every article was studied and reported independently; and in the event that one of the authors rejected the material, disagreements were resolved through consensus in a panel of 3 authors (the percentage of agreement was 100%). Methodological quality of selected articles was assessed using the Critical Appraisal Skills Programme (CASP). This instrument includes 12 questions about diagnostic tests developed by Jaeschke, Guyatt and Sackett in 1994 (18). Studied articles were divided into 3 categories (high quality, moderate quality and low quality). Those articles that were studied by CASP criteria and were categorized as moderate or high quality were used in this systematic review. All 11 remained articles were scored as moderate or high quality as they offered a comprehensive test for the direct assessment of children's ToM knowledge, in which they evaluated precisely stages of validities and reliabilities of the tests or mothers' preferences for introducing and elaborating on mental states in conversation with their young children (15, 17, 19-21).

***Data Extraction***

Data were extracted by two researchers (SZZA, ShJ) based on a previously prepared data extraction form, and differences were resolved by consensus in a panel of 3 authors. We studied full texts of the final articles and extracted some information about psychometric characteristics, validity and reliability of ToM tests. Characteristics of each test included the number of items/questions, population, time of test, dominance, mode of presentation, mode of responses, range of scores and type of scale used to score the items. Also, in this study, we studied face validity, content validity, criterion-related validity (concurrent and predictive validity), construct validity (convergent and divergent validity) and discriminate validity. To evaluate reliability, we studied intra-rater, inter-rater and test-retest reliability (for details see 22-25). Reliability and validity levels are expressed by correlation coefficients: Pearson correlation coefficient (r); Spearman correlation coefficient (P); Somer correlation coefficient (d); Intraclass correlation coefficient (ICC, Kappa value) and Cronbach's alpha (α) (22-25).

## Results

***Description of the Tests***

In the following, we have described the characteristics of 11 ToM tests that were identified after our systematic literature review (Tables 1 and 2). We identified some characteristics of these tests such as number of items/questions, population, time of test, dominance, mode of presentation, mode of responses, range of score and type of scale used to score the items. 

Happe' (1994) developed an strange story test that included 24 short vignettes, each accompanied by a picture and two test questions (comprehension question and judgment question). There are 12 types of story comprised of lie, White lie, joke, imaginary, misunderstanding, persuasion, appearance/reality, figure of speech, sarcasm, and fail to recall, double bluff and contrary emotion. The range of scores was 0-24. This test was developed for 26 normal children, 10 adults, 24 autisms and 13 children with mental disorders (8.6-20.6 year-old) in London and lasts from 20 to 60 minutes (19).

 Muris and Steerneman et al. (1999 and 2002) developed a ToM test that included three subscales: ToM 1 included ToM precursors (recognition of emotions and pretense, 29 items); ToM 2 included the first magnifications of real ToM (first-order-belief and false belief, 33 items) and ToM 3 included more advanced ToM aspects (second-order-belief and humor, 16 items). Total ToM scores range was between 0 and 78. This test was developed for 82 normal children, 20 children with PDD and 32 Attention Deficit Hyperactive Disorder (ADHD) with 5 to 12 year-olds and lasts about 35 minutes. This test was developed in Netherlands (20).

Hughes & Adlam et al. (2000) re-examined the reliability of false-belief tasks, using more standard (puppet and storybook) procedures. Forty seven normal children (aged 4.6-5.1 year-olds) participated in this study. They distinguished between five ‘standard’ and four ‘advanced’ theory-of-mind questions. In total, each child was presented with a maximum of nine test questions across six puppet or storybook tasks. The range of scores for standard and advanced tasks was 0-36 and 0-45, respectively. This test was carried out in London (17).

Peterson and Slaugheter (2003) developed the Maternal Mental State Input Inventory (MMSII) that was created to measure mothers' preferences for introducing and elaborating on mental states in conversation with their young children. Sixty one normal preschool (aged 4-5.7 year-olds) children and their mothers participated in the study. This questionnaire consisted of 12 stories. The instrument depicted episodes of every day family interaction (e.g., cooking, wrapping birthday presents). There are 4 response choices given with each story: Elaborated mental state (EMS), elaborated non-mental state (ENMS), non-elaborated mental state (NEMS) and non-elaborated non-mental state (NENMS). The total scores range was from 12 to 48; this test was carried out in Australia (26).

Wellman and Liu (2004) developed simple ToM tasks. Seventy five normal children (aged 2.11 to 6.6 years) were tested on 7 tasks that included various desires, diverse beliefs, knowledge access, content false belief, explicit belief, belief emotion and real-apparent emotion. In each task, there were two important questions that had to be responded verbally: A target question about the protagonist's mental state or behavior and a contrast or control question about the reality or another person’s state. This test was carried out verbally in Michigan and its range of scores was 0-14 (21).

Blijd-Hoogewys et al. (2008) developed a ToM Storybook. There are six color storybooks in total: How is Sam feeling? Sam goes to the park; Sam goes swimming; Sam visits his grandparents; Sam at the farm; and Sam's birthday. The test took 40-50 minutes to complete and was carried out verbally. There are 34 tasks that included various emotions, beliefs, desires and mental-physical distinctions. The 34 tasks consist of 92 questions: 74 ‘test questions’ and 18 ‘justification questions’ in total. Test questions were scored by 0-1 points and the total score was 74, and justification questions were scored by 0-1-2 points and the total score was 36. This test was done on 324 normal and 30 PDD-NOS children and was developed in Netherlands (15).

Hutchins and et al. (2008) developed a ToM test that referred to as Perceptions of Children's Theory of Mind Measure-Experimental Version (PCToMME). This test consists of 33 statements that the respondents should fill out in a form that accompanied by a response continuum of 20 metric unites. The measure was an index of caregivers' perceptions of children's ToM knowledge. Tasks were desire, pretense, intentionality, reality-appearance distinction, causes of emotions, mental-physical distinction, first-second order thinking, visual perspective-taking, affective recognition, empathy, social and logical inference, speech act, comprehension and production of mental state terms. Twenty parents and their children who had Autism spectrum disorder (ASD) (aged 4-12 year-old) and sixty normal children participated in this study (27).

O'Hare, Bremner et al. (2009) used Happe's strange story test (1994). One hundred forty 5-12 year-old children participated in the study. They used 12 strange stories: Lie, white lies, misunderstanding, sarcasm, persuasion, contrary emotions, pretend, joke, figure of speech, double bluff, appearance/reality and fail to recall. The range of scores was 0-24 (28).

Hutchins et al. (2012) developed a new version of Perceptions of Children's Theory of Mind Measure-Experimental Version (PCToMME) that was referred to as Theory of Mind Inventory (ToMI). It consisted of 48 statements accompanied by a response continuum of 20 metric units. Tasks were humor, sarcasm, counterfactual reasoning, distinction between jokes and lies, knowing and guessing, and understanding the mind as an active interpreter. This test was developed for 2-12 year-old ASD and normal children (135 ASD and 124 normal) (29).

 In Iran, Mohammadzadeh, Tehrani-doost and Khorrami (2012) assessed theory of mind skills of hundred 7-9 year-old primary school children by Moving Shapes Paradigm (behavioral tasks). Two kinds of animations were designed: 1- Random move sequence in which triangles move around the screen without any goal; 2- ToM sequence in which the triangles interact with each other. 

**Table 1:** Description of Psychometric Properties of ToM Tests

	**Not-reported**	**1-20 min**	**20-60 min**	
**Time of Tests**	3*,4,5,7,8,9	10, 11	1, 2, 6	
	**Pictorial story**	**Pictures and Figurine**	**Questionnaire**	**Computerized**
**Mode of Presentation**	1,2,3,6,8	5	4,7,9,11	10
	**Verbal**	**Forced-Choice**	**Filled by Parents**	
**Mode of Response**	1,2, 5,6,8,10	3,4	7,9, 11	

* These are the number of articles

**Table 2:** Description of Psychometric Properties of ToM Test

**Authors**	**Year**	**Number of questions**			**population**					**scoring**	**Scale of scoring**
		**question**	**story**	**animation**	**normal**	**disorder**	**Ages(years)**	**girl**	**boy**		
1. Happe'	1990	24	12	-	36	37	8.6-20.6	-	-	0-24	0-1
2. Muris & Steerneman	1999	78	9	-	82	52	5-12	24	46	0-78	0-1
3. Hughes & Adlam et al	2000	9	6	-	47	-	4.6-5.1	24	23	0-45	0-4 & 0-5
4. Peterson et al	2003	-	12	-	61	-	4-5.7	35	26	12-48	1-4
5. Wellman & Lui	2004	14	-	-	75	-	2.11-6.6	42	33	0-14	0-1
6. Blijd-Hoogewys et al	2008	92	6	-	324	30	3-12	157	167	0-110	0-1-2 & 0-1
7. Hutchins et al	2008	33	-	-	60	20	2-12	-	-	0-20	0-20
8. O'Hare, Bremner et al	2009	24	12	-	140	-	5-12	71	69	0-24	0-2
9. Hutchins et al	2012	44	-	-	124	135	2-17	-	-	0-20	0-20
10. Mohammadzadeh et al	2012	3	-	3	100	-	7-9	-	100	0-5	0-5
11. Tahiroglu et al	2014	42	-	-	456	-	2-7	239	226	-	0-4

**Table 3:** Description of Validities of ToM Tests

**Authors**	**Year**	**Types of Validity**				
		**Face/Content Validity**	**Construct Validity**		**Criterion-Related**	**Discriminate**
			**convergent**	**divergent**		
1. Happe'	1990	Not-reported	Not-reported	Not-reported	Not-reported	Not-reported
2. Muris & Steerneman et al	1999	High	0.80		Not-reported	0.24-0.58
3. Hughes & Adlam et al	2000	Not-reported	Not-reported	Not-reported	Not-reported	Not-reported
4. Peterson et al	2003	Not-reported	Not-reported	Not-reported	Not-reported	Not-reported
5. Wellman & Lui	2004	Not-reported	0.96		Not-reported	Not-reported
6. Blijd-Hoogewys et al	2008	0.248-0.454	0.26-0.79	0.41-0.43	Not-reported	Not-reported
7. Hutchins et al	2008	Reported	0.61-0.73		0.61-0.73	Not-reported
8. O'Hare, Bremner et al	2009	Not-reported	Not-reported	Not-reported	Not-reported	Not-reported
9. Hutchins et al	2012	Not-reported	0.66 & 0.72		0.73 & 0.82	Not-reported
10. Mohammadzadeh et al	2012	Not-reported	Not-reported	Not-reported	Not-reported	Not-reported
11. Tahiroglu et al	2014	Not-reported	0.28 ≤r≥ 0.31		Not-reported	Not-reported

**Table 4:** Description of Reliabilities of ToM Tests

**Authors**	**Year**	**Reliabilities**				
		**Test-Retest**	**Number of Apart**	**Internal Consistency**	**Inter-Rater**	**Number of Raters**
1. Happé	1990	Not-reported	Not-reported	Not-reported	92-100 %	2
2. Muris & Steerneman et al	1999	0.99	8 weeks	0.92	>0.87	2
3. Hughes & Adlam et al	2000	0.66 & 0.77	4-6 weeks	0.66 & 0.88	Not-reported	Not-reported
4. Peterson et al	2003	Not-reported	Not-reported	0.72	Not-reported	Not-reported
5. Wellman & Lui	2004	Not-reported	Not-reported	0.96	Not-reported	Not-reported
6. Blijd-Hoogewys et al	2008	0.86 & 0.98	2-3 weeks	0.47-0.90	0.90-0.97	5
7. Hutchins et al	2008	0.89-0.98	1 week & 3.5 Months	Not-reported	Not-reported	Not-reported
8.O 'Hare, Bremner et al	2009	Not-reported	Not-reported	Not-reported	0.66 & 0.100	3
9. Hutchins et al	2012	0.89	12-78 days	0.98	Not-reported	Not-reported
10. Mohammadzadeh et al	2012	Not-reported	Not-reported	Not-reported	Not-reported	Not-reported
11. Tahiroglu et al	2014	0.88	1-4 weeks	0.89-94	Not-reported	Not-reported

The ToM sequence type presented 3 animations to assess ToM (coaxing, hide and seek and surprising animation). Each animation lasted 34-45 seconds. The answers were verbally evaluated in four dimensions: Intentionally (degree of mental states attribution and ToM related vocabulary 0-5 points), appropriateness of description (degree of correctness of answers 0-3 point), length of answers (0-4 point), and using emotional words (30). 

Tahiroglu et al. (2014) developed the Children’s Social Understanding Scale (CSUS), a parent-report ToM measure in North America. The 42-item final version of the CSUS consisted of approximately equal numbers of items (7 items) in each of its six subscales (i.e., belief, knowledge, perception, desire, intention, and emotion) that were filled by 465 parents of 2 and 7 year old children. Parents were asked to rate their children on a 4-point Likert scale. The CSUS took about 20 minutes (31).

***Validity and Reliability***

Tables3 and 4 demonstrates the types of validity and reliability of ToM tests. In the following, we present the methods of validity and reliability used in each test separately: 

Happe' (1994) found that three groups of children (autism, mental handicapped and normal children) differed significantly in total ToM scores, with autistic subjects scoring the least. They expressed that this supports the validity of the ToM tasks. The degree of concordance in inter-rater reliability ranged from 92 to 100%. They showed that this test can discriminate between normal children and autistic and mentally retarded children (19).

Muris and Steerneman et al. (1999) studied three types of validity (construct, concurrent and discriminate) and reliability (test-retest, internal consistency and inter-rater) for their ToM test. The construct validity was 0.80. For concurrent validity, the Pearson correlation coefficient (r) between ToM test and other tests were calculated that was significant (0.37≤r≤0.77). For discriminate validity, correlation between age and ToM (r = 0.24) and ToM and total IQ (r = 0.58) was calculated. For test-retest reliability, intraclass correlation coefficient, 8 week apart, was calculated (ICC = 0.99). Internal consistency of ToM test was calculated by Cronbach alphas (α = 0.92). Also, inter-rater reliability of ToM test with two raters by Kappa's scale was larger than 0.87 (20).

Hughes, Adlam et al. (2000) studied test-retest reliability and internal consistency. Pearson correlation for test-retest reliability of standard and advanced false belief tasks 4-6 week apart was 0.77 and 0.66, respectively. Also, total Cronbach alphas for the internal consistency of their test were 0.88 and 0.66, respectively (17). 

Peterson and Slaughter (2003) reported internal consistency of their parent-reported MMSII questionnaire. Cronbach's alphas for the elaborated mental state (EMS) total score were 0.72 and for the ENMS, NEMS and NENMS totals were 0.61, 0.65 and 0.62, respectively (26).

Wellman and Liu (2004) had a report on scaling 7 ToM tasks. Two methods for scale analysis (Guttman scaling or scalogram analysis and Rasch Model) were used. The responses of 80% of the children (60 of 75) fit five-item Guttman scale. The coefficient of reproducibility from a scalogram analysis of these data was 0.96. Also, Rasch model showed that their 7 item tests fit a single scale construct. Moreover, the relationship between age and Guttman scale score and Rasch Model was also calculated (r = 0.64) (21).

Hutchins et al. (2008) that developed PCToMM-E questionnaire reported criterion-based construct validity in normal children and in those with ASD. In the ASD group, the Pearson correlation was 0.61 between verbal mental age (VMA) and ToM tasks; Spearman correlation was 0.67 between PCToMM-E and ToM task; and Spearman correlation was 0.73 between the predictive measure of ToM abilities and the ToM tasks. In the normal group, Pearson’s correlation showed a signification relationship between child’s age and PCToMM-E score (r = 0.68). Also, the difference between judgments of ASD mothers and normal mothers about children was significant (P<0.01), supporting construct validity. For face validity, they followed the literature, and each item was developed so that it was a face valid indicator of child’s knowledge. To evaluate the convergent validity of the PCToMM-E, 12 of 16 items on a ToM task battery that were found to have good test–retest reliability were administered, and consisted of 12 test questions within seven tasks. Test-retest reliability in one week apart in ASD and normal children was 0.94 and 0.98, respectively and it was 0.89 in 3.5 month apart in ASD group (27). 

Blijd-Hoogewys, Greet, Serra and Minderaa (2008) studied two types of validity (content and construct validity) and three types of reliability (test-retest, inter-rater reliability and internal consistency). For content validity, they studied the correlation of subtypes of ToM in three groups that varied from 0.248 to 0.454. For construct validity, they tested both convergent and divergent validity of ToM storybooks. Concerning convergent validity, correlation with three similar tests was calculated and it was between 0.26 and 0.79. For divergent validity, correlations with language and intelligence tests were calculated by Pearson product-moment correlations, which were between 0.41 and 0.43. The test–retest reliability for normal children was 0.86 and it was 0.98 for PDD_NOS. Moreover, Cohen’s kappa scale was used to assess inter-rater reliability. The correlations between five raters were 0.90-0.97. For internal consistency, Cronbach’s alpha was used and correlation for age varied (0.47-0.80) and it was 0.90 for dichotomous items (15).

O'Hare, Bremner et al. (2009) studied only inter-rater reliability, in which correlation between three raters was 100% apart from the banana ‘pretense’ story where it was 66% (28).

Hutchins et al. (2012) evaluated test-retest reliability, internal consistency and criterion-related construct validity of ToMI. For test-retest reliability, Pearson’s product moment correlation was calculated for ASD and normal children, using an interval of 12–78 days (in both of group, r = 0.89). Internal consistency was assessed using Cronbach’s alpha (α = 0.98). Also, criterion-related validity was 0.73 and 0.82 in ASD and normal children, respectively. Construct validity was 0.66 and 0.72 in two groups, respectively (29).

Mohammadzadeh, Tehrani-doost and Khorrami (2012) had no report about the validity and reliability of their computerized test and just explained that their test was based on original ToM test developed by Castelli and Frith (2000) (30, 32). 

Tahiroglu et al. (2014) described cross-validation data for the CSUS in a different sample of preschool children with a different set of ToM tasks. Also, they studied internal consistency, test–retest reliability, and relation of the scale to children’s performance on other ToM tasks in three studies. Internal consistency was 0.94 and 0.89 for the full and short scales, respectively. Test–retest reliability was 0.88 with 1–4 weeks apart. In Study 1, correlation of the full and short scales to children’s performance on other ToM tasks was between 0.15 and 0.37, and most of them were significant. In Study 2, cross-validation data for the CSUS in a different sample of preschool children with a different set of ToM tasks was significant (0.22 ≤r≥ 0.47). In study 3, for further construct validity, the correlation of full and short scales and cognitive tests was between 0.31 and 0.28, respectively (31).

## Discussion

The aim of this study was to systematically review the validity and reliability of comprehensive theory of mind tests for normal preschool children. Many children with different language and cognitive disorders such as hearing loss, PDD, and SLI have problems with ToM tasks, particularly false belief tasks (33-42). Also, normal children may perform differently from one another in ToM tasks (33-41). The researchers confirmed that language and cognitive disorders are related to ToM deficits and that assessing these skills is of prime importance in children (33-41). The theory of mind is a cognitive concept (33-41). Therefore, all professionals that have researched the field of language and cognitive disorders (e.g., psychologists, neuropsychologists, and speech language pathologists) can benefit from the presented tests in this systematic review. 

From 1980 to 1990, most researchers used single task measurements (false belief tasks) to assess ToM (15). These assessments may be quick, but provide no information about other aspects of ToM (e.g., facial expression recognition, pretend plays, irony and hummer) and these aspects cannot evaluate these tasks (15, 31). Therefore, the researchers used more comprehensive instruments by means of multiple tasks (15). Such instruments can reduce standard errors and make measurements more reliable and valid. Therefore, the use of a comprehensive test can provide the researchers with the chance to compare different components of ToM based on developmental levels (15).

The literature indicated that most of ToM tests have been developed for school-age children and older, but the development of different ToM levels initiated from the age of 1-2 years and continued to adolescents (15). Therefore, it is better to start assessing this skill before school, and the type of task should be selected according to the age range. It seems that using difficult tasks such as irony and hummer comprehension for preschool children is not appropriate (15). In this study, the aim was to collect comprehensive ToM tests that included all different ToM components that were usable for preschool children. However, we found few tests with this condition (15, 20, 21and 28). Other studies focused on false belief tasks and/or were carried out using questionnaires completed by parents (17, 26, 27, 29 and 31). 

Two of the important characteristics in the reviewed ToM tests were the mode of presentation and the mode of children responses. Most of researchers presented tasks by visual-auditory stories to which children responded verbally (15, 17, 27-30). Reading storybooks provide a rich source of mentalizing information for children (15). Usually, these tests can be used for children with various language disorders, and this method of presentation can be used especially for mentally retarded children (15).

We defined some questionnaires in this review that were used to assess ToM. The literature showed that the use of informal measurement such as questionnaires that evaluate mother's perception from children's ToM knowledge can be an index related to children's ToM abilities (26, 27, 29 and 31). For example, if parents suspect that children's ToM abilities are high, perhaps, the children's ToM scores will be high in ToM tests as well (26, 27, 29 and 31). However, these tests are based on knowledge of other important individuals in children's life not on the real abilities of children (26, 27, 29 and 31). In this review, we found that Hutchins et al. (2008 & 2012) and Peterson et al. (2003) and Tahiroglu et al. (2014) developed questionnaires to assess children's ToM that was filled by parents (26, 27, 29 and 31). Each of them reported validity and reliability of their questionnaires. However, these tests did not directly evaluate ToM levels in children.

The various types of validity and reliability of ToM tests were reported in some of these studies. Most studies evaluated construct validity, test-retest reliability and internal consistency. For example, In Happé's and O'Hare's studies inter-rater reliability was only reported (19, 28). Peterson et al. (2003) studied only internal consistency and Wellman and liu (2004) studied construct validity and internal consistency (21, 26); Hughes and Adlams (2000) reported test-retest reliability and internal consistency (17); Hutchins et al. (2008 and 2012) and Tahiroglu et al. (2014) reported construct validity, test-retest reliability and internal consistency (27, 29, 31) and finally Mohammadzade et al. (2012) developed a computerized ToM test for the first time in Iran which took a short time to complete, but they had not reported the validity and reliability of their test (30). Some articles also offered strong evidence for other types of validity and reliability (15, 20). Murris and Steerneman (1999) studied three types of validity (construct, concurrent and discriminate) and three types of reliability (test-retest, internal consistency and inter-rater) (20). Also, Blijd-Hoogewys et al. (2008) studied two types of validity (content and construct) and three types of reliability (test-retest, internal consistency and inter-rater) (15). In both studies, the reported validity and reliability were perfect, but there were differences between these studies. ToM is a cognitive and abstract concept, and perhaps rater's ideas can influence its scoring. Therefore, it is better to evaluate inter-rater reliability. The most number of raters was found in Blijd-Hoogewys's study. Also, the numbers of normal population in Blijd-Hoogewys's study was 324 versus 82 in Murris and Steerneman's study. The age range in these studies was different too. It was 3-12 year-olds in Blijd-Hoogewys's study and 5-12 year-olds in Murris and Steerneman's study. Because most preschool children are younger than 5 years old, it seems that Blijd-Hoogewys's test is easier for them. 

In the past years, the development of ToM tests started and evolved slowly. Each of the tests was developed for special aims and groups. This systematic review from ToM tests can give useful information about theory of mind tests. In addition, it can help the researchers and clinicians to select their ToM tests based on their clinical or research aims. For example, if the aim is research, using valid and reliable tests is an advantage and if the aim is clinical, using tests that have easy presentation or have short time such as questionnaires and computerized tests can be useful.

## Limitations

Although we have tried to collect the most relevant data for our study, focusing only on the published English articles with limited Keywords was one limitation of this study which could affect the results. Further investigation on unpublished and other languages data is necessary to reach a better estimation of child ToM tests. Also, many ToM tasks were found from 1980 to 1990, but most of them had used single task measurements to assess ToM. Also, most of new ToM tests were developed for school-age children and older ones. In this systematic review, we focused just on comprehensive ToM tests for normal preschool children from 1990 to June 2015. Therefore, investigation on all types of ToM tasks and tests for all age ranges is necessary.

## Conclusion

According to this review, the defined ToM tests were different in populations, tasks, mode of presentations, scoring, mode of responses, times and other variables. Also, they had various validities and reliabilities. Therefore, it is recommended that the researchers and clinicians select the ToM tests according to their aims and psychometric characteristics, validity and reliability of these tests.

## Appendix 1

The search strategy was modified according to each database’s specifications. In the following paragraphs the strategy used for Pubmed Medline is shown as an example:

#1. ‘Theory of mind’ [mesh] AND test AND children.
